# Whole-loop mitochondrial DNA D-loop sequence variability in Egyptian Arabian equine matrilines

**DOI:** 10.1371/journal.pone.0184309

**Published:** 2017-08-31

**Authors:** William Hudson

**Affiliations:** William Hudson MD, Hudson Cardiology P.C., Atlanta, Georgia, United States of America; The University of Melbourne, AUSTRALIA

## Abstract

**Background:**

Egyptian Arabian horses have been maintained in a state of genetic isolation for over a hundred years. There is only limited genetic proof that the studbook records of female lines of Egyptian Arabian pedigrees are reliable. This study characterized the mitochondrial DNA (mtDNA) signatures of 126 horses representing 14 matrilines in the Egyptian Agricultural Organization (EAO) horse-breeding program.

**Findings:**

Analysis of the whole D-loop sequence yielded additional information compared to hypervariable region-1 (HVR1) analysis alone, with 42 polymorphic sites representing ten haplotypes compared to 16 polymorphic sites representing nine haplotypes, respectively. Most EAO haplotypes belonged to ancient haplogroups, suggesting origin from a wide geographical area over many thousands of years, although one haplotype was novel.

**Conclusions:**

Historical families share haplotypes and some individuals from different strains belonged to the same haplogroup: the classical EAO strain designation is not equivalent to modern monophyletic matrilineal groups. Phylogenetic inference showed that the foundation mares of the historical haplotypes were highly likely to have the same haplotypes as the animals studied (p > 0.998 in all cases), confirming the reliability of EAO studbook records and providing the opportunity for breeders to confirm the ancestry of their horses.

## Introduction

Arabian horses are one of the oldest pure breeds [1, 2]. In 1908, the Egyptian Agricultural Organization (EAO) established a closed breeding program of Arabian mares and stallions, known as Egyptian Arabian horses, which have been maintained in a state of genetic isolation since the mid 20^th^ century. Up to fifteen generations of EAO Egyptian Arabian pedigree horses are documented, and the EAO keeps accurate records of the pedigrees of its stock organized by matriline (dam line), with each matriline having a unique family name, known as its strain (rasan). Traditional breeders (the Bedouin) have maintained strictly separated rasans and avoided crossbreeding between Arabian and non-Arabian horses; as a consequence, individuals within a rasan should belong to the same matriline and have the similar mitochondrial DNA (mtDNA) haplotype.

mtDNA sequencing has been extensively used to study intra- and inter-species genetic relationships and structure since it is maternally inherited, haploid, and not subject to genetic recombination. In particular, the D-loop hypervariable region (HVR) of mitochondrial DNA yields the most information in matrilineal studies since it possesses a high degree of sequence variation but only a moderate mutation rate. D-loop mtDNA sequencing has previously been used to evaluate matrilineal pedigree validity in Polish Arabians, Lippizans, and English Thoroughbreds [3–8]. The D-loop region in horses contains two highly variable segments (HVR1 and HVR2), four conserved blocks (CSB), and variable eight base pair repeats [9].

Only a few previous mtDNA sequencing studies have included Arabian horse pedigrees [2, 6, 7, 10–12], and most of these only sequenced about 400 base pairs (bps) of the 1200 bp mtDNA D-loop. Furthermore, only a few of the EAO historical families have been haplotyped. An analysis of the entire mtDNA D-loop sequence of horses descended from all of the extant historical families of the EAO horse-breeding program was therefore conducted to investigate their evolutionary origins, within-population genetic diversity, and to better understand how historical strain breeding relates to maternal genetics.

## Material and methods

### Study population and sample collection

One hundred and twenty-six horses were included in the study. Study subjects were not chosen at random but were selected from living matrilineal descendants of the EAO foundation mares in the published pedigrees of Arabian horses registered with the Arabian Horse Association (AHA); the horses’ names, pedigrees, AHA registration number, coat color, and markings are on record.

#### Polymerase chain reaction (PCR) amplification, whole D-loop sequencing, and data analysis

Primers to amplify the whole D-loop region of the horse mitochondrial genome (reference sequence X79547) were used as previously described [6]. Primers were designed to minimize the possible amplification of nuclear mtDNAs (NUMTs) that might overlap with the D-loop [6]. The HVR1 primer set was used to amplify the region upstream from sites 15440 to 16108 (forward: 5′-AGCTCCACCATCAACACCCAAA-3′, reverse 5′-CCATGGACTGAATAA CACCTTATGGTTG-3′), while the HVR2 primer set was used to amplify the region downstream from 16377 to 16642 (forward 5′-ACCTACCCGCGCAGTAAGCAA-3′, reverse 5′-ACGGGGGAAGAA GGGTTGACA-3′). PCR for HVR1 and HVR2 was conducted on each DNA sample in a final volume of 30 μl with the following reagents: 0.2 mM dNTPs, 0.5 μM of each primer, 2.5 mM MgCl_2_, 1X PCR buffer, 1U Titanium Taq polymerase (Clontech, Mountain View, CA), and 5 μl of eluted DNA. Cycling conditions for both PCR reactions were as follows: denaturation at 95°C for 5 min; 35 cycles of 95°C for 30 s, 58°C for 30 s, and 72°C for 1 min; extension at 72°C for 7 min; hold at 10°C. PCR products were purified using a 1:1 Agencourt-XP purification according to the manufacturer’s instructions (Beckman Coulter Inc., Pasadena, CA).

Forward and reverse sequencing was performed using the BigDye^®^ Terminator v3.1 Cycle Sequencing Kit (Applied Biosystems, Foster City, CA). Sequencing products were purified using SEPHADEX^®^ G-50 medium grade (GE Healthcare Life Sciences, Pittsburgh, PA) before being analyzed using a 3730xl capillary sequencer (Applied Biosystems) and edited using Geneious version 6.1.7 (Biomatters Ltd., Auckland, NZ). Edited sequences for each region (HVR1 and HVR2) were aligned to the reference sequence (X79547) using the MUSCLE algorithm included in the Geneious software package. The aligned sequences were then concatenated to form continuous coverage of the D-loop for each sample. The unsequenced non-hypervariable region from 16109–16376 was filled with sequence gaps (-) to maintain the correct segment length and sequence neutrality for that region. Concatenated sequences were separately re-aligned to X79547 using the previous parameters. MEGA version 6.0.5 was used to generate a variable site table for the sample lineages, as previously described [13]. All DNA sequences are available in GenBank under accession numbers KU569909-KU569917 and KU575096.

### Statistical analysis

The sequencing results were first examined for discordant haplotypes, which were defined as any haplotype that occurred only once in the study population and was unique; a discordant haplotype was interpreted as indicating that the sample was either obtained from the incorrect horse or that the horse’s pedigree was incorrect. No attempt was made to analyze pedigree errors since this was not the primary objective of this study.

Phylogenetic inference was performed using MrBayes version 3.2. Input files were prepared using R (version 2.14.1). To calculate the measure of uncertainty, the Huelsenbeck and Bollback approach for inferring ancestral states was used [14], implemented in MrBaynes, and the validity of the haplotype identity inferences was addressed using the General Time Reversibility Model [15]. The Genbank control reference sequence X79547 (‘a Swedish horse’, as used previously [6]) was used as an out-group to root the tree. Only the terminal pedigree line individuals could be used in the analysis. In cases of direct mother-daughter or direct grandmother-granddaughter links occurring in the study subjects, the proximal individual was deleted from the statistical analysis and the distal horse was used. The program required a minimum of four individuals per historical family (HF), and those historical families with less than four individuals were excluded from the analysis.

## Ethics statement

All horse owners provided written informed consent after full explanation of the purpose and procedures of the study. Venous blood samples were obtained directly from the owners as part of the horses’ routine surveillance and care and preserved on Whatman FTA Elute Micro Cards (GE Healthcare Life Sciences, Pittsburgh, PA). The study protocol was not submitted to or approved by an institutional animal care and use committee (IACUC), but the research was conducted in full consideration of, and adherence to, the Basel Declaration.

## Results and discussion

All horses shared a haplotype with at least one other horse. Since the majority of previous Arabian horse mtDNA studies only examined HVR1, polymorphisms occurring in this region were first examined for comparison with previous studies and evolutionary data. After eliminating four mutational hotspots at 15585, 15597, 15604, and 15650 [8, 16], 126 samples representing 14 matrilines provided evidence for nine unique whole loop mtDNA haplotypes based on HVR1 analysis alone (Table 1; designated M1-M10, with M1 and M3 the same in HVR1 polymorphism analysis). We next sought to establish whether these haplotypes corresponded to previous haplotype/haplogroup designations from evolutionary studies based on the nomenclature introduced by Cieslak et al. (2010) (haplogroups denoted A to K and X1 to X8). Eight out of the nine haplotypes corresponded to ancient haplotypes defined by Cieslak et al. (Table 1) and were similar in distribution to those recently reported for Arabian horses by Moridi et al. [17] (haplogroups A, D3, F, I, K3, X2, X3); one haplotype could not be assigned (M9). The wide variety and similarity of haplogroups within the EAO historical families is consistent with the large number of haplotypes known to occur in domestic horses and likely to have arisen from ancestral variability from primitive breeds across wide geographical areas over the last few thousand millennia [16]. For instance, haplogroup A has been detected in ancient remains of pre-domesticated horses from North East Siberia and Eurasia dating to the Late Pleistocene period (approximately 12,000 B.C.) and haplogroup F from the Copper Age. Three of the nine haplotypes had previously been reported in other Arabian horse studies (Table 1); El Dahma (M1, equivalent to A12 in [12]), Jellabiet Feysul and Ghazieh (M4, equivalent to A11 in [12] and M in [7]), and Rodania (M5, equivalent to A01 in [12] and K in [7]). These data suggest that the original horses captured and domesticated by the Bedouin already harbored a large amount of genetic diversity of Eurasian origin.

**Table 1:** EAO historical family foundation mares, whole loop mtDNA haplotypes.

Founding mare of each historical matriline	Date of birth	Coat color	Pedigree	Strain designation	Name of breeder	Previously studied	Provenance	Number of horses studied/available for inference	P (Founder haplotype)	Haplotype	Haplotype (haplogroup) (Cieslak et al. 2010)
El Dahma	ca. 1879	Unknown	Unknown	Dahmah Shahwaniyah	Ali Pasha Sherif	Yes	Unknown	23/19	0.999	M1	(D)3
Bint El Bahreyn	1898	Bay	Unknown	Dahmah Shahwaniyah	Aissa Ibn Khalifa, Sheikh of Bahrain	No	Unknown	9/8	0.998	M2	(I)
Roga El Beida (Roda)	1882	Grey	Unknown	Saqlawiya Jedraniyah	Ali Pasha Sherif	No	Unknown	4/4	0.999	M3	(D)3
Ghazieh I	Prior to 1860	Unknown	Unknown	Saqlawiya Jedraniyah	Ibn Sudan of the Ruala	Yes	Unknown	13/8	0.999	M4	(X3)c1
Jellabiet Feysul	Unknown	Unknown	Unknown	Saqlawiya Jedraniyah	Feysul Ibn Turki	No	Unknown	7/6	0.999	M4	(X3)c1
Venus (Yunis)	Prior to 1893	Chestnut	Unknown	Hadba Enzahiyah	Unknown	No	Imported to Egypt by Hassan Abu Amin’s Agha in 1893	12/11	0.999	M5	(K3)
Rodania	Ca. 1869	Chestnut	Unknown	Kuhayla Rodan	Ibn Rodan of the Ruala	Yes	Unknown	11/9	0.999	M5	(K3)
Bint Karima	1935	Grey	Rasheed x Karima (Blunt)	Unknown	Unknown	No	Purchased from Kafr Ibrash Farm	2/2	Too few horses	M5	(K3)
Hind	1942	Grey	Sire: Obeyan El Safi	Saqlawiya	King Ibn Saoud	No	Unknown	7/7	0.999	M5	(K3)
El Obeya Om Grees	Unknown	Unknown	Unknown	Abbeya Om Jurays	King Ibn Saoud	No	Unknown	7/5	0.999	M6	(A)
El Kahila	1921	Dark bay	Unknown	Kuhayla Krush	King Ibn Saoud	No	Unknown	11/10	0.999	M7	(X3)
Mabrouka	1930	Bay	Unknown	Saqlawiya	King Ibn Saoud	No	Unknown	8/8	0.999	M8	(F)
Nafaa	1941	Roan	Unknown	Koheila	King Ibn Saoud	No	Unknown	5/3	Too few horses	M9	No designation
El Samraa	1924	Grey	Hab el Rih x Bint El Sheikh	Unknown	Unknown	No	From Sheikh Omar Abdel El Hafiz	7/7	0.999	M10	(X2)

Analysis of the whole D-loop sequence yielded additional information compared to HVR1 analysis alone, with 10 haplotypes detected in total from 42 polymorphic sites compared to 9 haplotypes from 16 polymorphic sites, respectively (Table 2). There was also one cytosine insertion in haplotype M9 at base position 16371. Five of the ten haplotypes had unique substitutions (M2, M4, M5, M8, and M10). Although π decreased from 0.0155 to 0.0094, the haplotype diversity remained static and relatively low at 0.85 (HVR1) and 0.86 (whole-loop) (Table 3). Khanshour and Cothran (2013) reported similar whole-loop π values in Arabian horse populations (0.010–0.017), which are lower than the nucleotide diversities reported in Iranian (0.02) [17] and Chinese breeds (0.02) [18]. In spite of the historical origins of the maternal lineage, this lower nucleotide diversity is likely to reflect the careful selection of particular phenotypic characteristics of these horses and closed breeding programs.

**Table 2:** A variable base position table demonstrating the ten whole loop mtDNA haplotypes found in subjects. The region corresponding to HVR1 is shown in grey. Blank spaces indicate concordance with the X79547 GenBank sequence.

**Haplotype**	**15494**	**15495**	**15496**	**15534**	**15538**	**15542**	**15601**	**15602**	**15603**	**15635**	**15649**	**15666**	**15667**	**15703**	**15709**	**15720**	**15771**	**15777**	**15806**	**15807**	**15809**
**Ref**	T	T	A	C	A	C	T	C	T	C	A	G	A	T	C	G	C	A	C	C	A
**M1**		C										A				A					
**M2**		C			G			T							T	A	T				
**M3**		C										A				A					
**M4**		C				T		T		T		A		C		A					
**M5**		C						T					G	C		A	T	G			G
**M6**		C						T								A					
**M7**		C				T		T		T		A				A					
**M8**		C					C	T								A	T		T		
**M9**		C			G			T								A	T				
**M10**	C	C	G	T				T	C		G					A	T			T	
**Haplotype**	**15826**	**15827**	**15838**	**15869**	**15870**	**15871**	**15956**	**15974**	**16009**	**16033**	**16040**	**16057**	**16068**	**16071**	**16079**	**16371**	**16442**	**16543**	**16546**	**16559**	**16635**
**Ref**	A	A	C	C	C	C	A	C	T	T	A	A	T	T	A	T	C	T	T	C	A
**M1**	G											G				C					
**M2**	G				T			T								C	T				
**M3**												G				C					
**M4**					T					C						C					
**M5**							G				G					C		A			G
**M6**	G				T		G	T								C					
**M7**					T											C					
**M8**		G	T	T			G		C					C		C		A	C	T	G
**M9**	G							T								C					
**M10**					T	T	G	T					C		G	C					

**Table 3:** HVR1 vs. whole-loop indices.

	Number of haplotypes	Haplotype diversity	Nucleotide diversity	Average nucleotide differences
HVR1	9	0.85 (0.015)	0.0155 (0.0005)	3.6
Whole-loop	10	0.86 (0.014)	0.0094 (0.0003)	9.6

These data confirm that modern mtDNA haplotyping does not correspond to the traditional strain designation based on Bedouin breeding traditions that depend on maternal lineage [6, 12]. Although all sample horses in each historical family possessed the same haplotype, haplotype M4 contained individuals from two historical families (Ghazieh and Jellabiet Feysul), while haplotype M5 contained individuals from four historical families (Venus, Rodania, Bint Karima, and Hind). This can be explained by two mechanisms: either all the historical families originated from a single common distant ancestress, or the results could be due to pedigree errors. The latter is unlikely since this would require three pedigree errors, and the former is supported by haplotype similarities with ancient haplogroups. Furthermore, individuals from different strains belonged to the same haplogroup (El Dahma and Roga El Beida to D3a3). Members of the Ghazieh and Jellabiet Feysul historical families had the same whole loop mtDNA haplotype and appeared to belong to a single matriline, which is consistent with previous results [12].

The aim of selective mating is to produce offspring with certain desirable phenotypic or behavioral characteristics. Genetic analysis can help clarify identification and the parentage of horses. Phylogenetic analysis showed that the foundation mares were highly likely to have the same haplotypes as the animals studied (p > 0.998 in all cases, Table 1), confirming the reliability of EAO studbook records; sequence stability ensures reliable determination of maternal haplotypes through multiple maternal generations over the timeframe of studbook records. These data constitute a valuable database against which living horses can be compared. The mares from which the EAO matrilines were derived were bred by Bedouin tribes living on the Arabian peninsula and have been sold to foreign buyers for several centuries, often with little or no documentary evidence of their pedigrees. If an owner suspects that their horse is descended from EAO matriline rootstock, DNA can be tested and the haplotype compared to these data to confirm the accuracy of the matrilineal pedigree.
